# On the Treatment of Fracture of the Radius at the Styloid Process by Means of Gordon’s Splint

**Published:** 1866-11

**Authors:** Lawson Tait


### On the Treatment of Fracture of the Radius at the Styloid Process by means of Gordon's Splint.

BY LAWSON TAIT, ESQ.

(From Braithwaite's Retrospect, July, 1866.)

Few fractures have had so many ingenious splints devised for their treatment as that known as Colles' fracture, yet most or all have been given up, and the ordinary palmar and dorsal straight splints generally used, although frequently with unsatisfactory results.

In spite of the utmost care most cases of this fracture turn out unsatisfactorily, and many are the actions of damages that have been raised on its account. The reason of this non-success is, I think, very plain; let any one examine his own wrist, and the following explanation will be clear. Holding the hand straight out in a plane with the forearm, it will be seen that, while the dorsal aspect is almost a straight line, there is considerable concavity at the wrist on the palmar aspect; indeed, that a line drawn from the elbow to the ball of the thumb would be, so to speak, the chord of a segment of a circle. Thus it is that when an arm, with the radius broken as it is in Colles' fracture, is pressed by two straight splints, one on either aspect, extending from the elbow to the fingers, the upper fragment must necessarily be pressed towards the palmar aspect of the limb; while the lower fragment, which is practically the same in this condition as the ball of the thumb, is pressed in the opposite direction—in fact, that the distortion is only increased by the splints, as they press the fragments in the very direction in which they are already displaced. If this be correct, then it is easy to understand the success which has attended the use of Dr. Gordon's splint in the treatment of this fracture, and to believe that it is devised on sound anatomical and mechanical principles—that it really is what all splints ought to be, viz: a dermal skeleton.

This instrument was originally invented and described by Dr. Gordon, of Belfast; the only notice, however, which I am aware that it has subsequently received is in a paper by Mr. Stokes in the Dublin *Medical Journal.* It is composed of two pieces of wood, the one for the palmar aspect of the forearm being about nine inches long, two and a quarter inches wide at the wrist, and three and a half wide at the elbow; the surface to be in contact with the skin is slightly hollowed out to fit the arm, and along its radial border it has screwed to it a wooden bar or pad, which is rounded off at the ditsal extremity to fit the concavity of the radius; this latter, of course, necessitates that, to fullfil this condition, separate splints are required for the right and left arms.

The pad, in addition to its being rounded off at the extremity, is rounded all along its inner surface so as to press separately against the radius throughout nearly its whole length, and it is of sufficient height to embrace rather more than half the thickness of the forearm. The other portion of the apparatus consists of a plain piece of three-eight inch board, two inches and a quarter broad, and two inches longer than its fellow; it is for application to the dorsal aspect of the forearm, and has the surface to be in contact with the skin slightly hollowed, and it likewise has its distal extremity transversely rounded. Its application is effected as follows:—The fracture having been reduced, the limb is retained in position by an assistant, the lower part of the apparatus is then applied, padded with spongiopiline or lint, to the radial portion of the forearm alone, and not to the hand. Then the upper splint is to be applied, likewise padded, in such a manner that the proximal ends of the two parts of the apparatus are maintained at the same level, while the distal end of the upper one projects about two inches beyond the end of the radius. For a more particular description and a drawing, see *Dublin Medical Journal*, for February, 1865. The whole apparatus is firmly secured by two small straps with buckles. In this manner no pressure is exerted on either of the fragments but what is calculated to keep them in their correct position. The arm, during the after progress of the case, is recommended to be kept in the position most agreeable to the patient, which will be found to be that of almost complete pronation. In the employment of this apparatus the wrist will be found to be confined only to a limited extent, while the movements of the fingers and carpo-metacarpal articulations are quite unimpaired; thus entirely doing away with the most objectionable condition of stiff joints, which is such an annoyance both to surgeon and patient for weeks after the common splints have been removed from the forearm.

